# A machine learning-based classification model for interstitial lung disease in rheumatoid arthritis

**DOI:** 10.3389/fmed.2026.1807594

**Published:** 2026-05-14

**Authors:** Mingyao Li, Qiaoli Wang, Junfeng He, Xia Wang, Yangyang Xu, Liwei Yang, Lin Feng

**Affiliations:** 1 Department of Rheumatology and Immunology, Deyang People’s Hospital, Deyang, Sichuan, China; 2 Health Management Center, Deyang People’s Hospital, Deyang, Sichuan, China

**Keywords:** CatBoost, interstitial lung disease, machine learning, rheumatoid arthritis, risk classification model

## Abstract

**Objective:**

This study aimed to develop and validate a preliminary classification and diagnostic model for rheumatoid arthritis-associated interstitial lung disease (RA-ILD) using routine, readily available clinical and laboratory parameters. Multiple machine learning algorithms were employed to construct a practical risk assessment tool suitable for use in primary hospital settings.

**Methods:**

Clinical data were retrospectively collected. Patients were divided into RA and RA-ILD groups. After preprocessing, the cohort was randomly divided into training and validation sets. Variables demonstrating a trend toward significance on univariate analysis were subjected to LASSO regression, and feature variables were ultimately identified. Five machine learning models were constructed: CatBoost, logistic regression, support vector machine, decision tree, and random forest. Model performance was assessed on the validation set using accuracy, precision, recall, F1 score, and the area under the receiver operating characteristic (ROC) curve (AUC). The SHapley Additive exPlanations (SHAP) framework was used to identify key features and quantify their contributions to the optimal predictive model.

**Results:**

A total of 410 patients with RA were enrolled, among whom 100 (24.39%) were diagnosed with RA-ILD. 23 variables with a trend toward significance on univariate analysis were subjected to LASSO regression. Finally, seven features were selected for model construction: age, smoking history, LYMPH, LDH, RF, CA125, and CA199. Based on these features, five machine learning models were established for RA-ILD classification. In the validation set, the CatBoost model achieved the highest AUC of 0.784 (95% CI: 0.656–0.885) and the lowest Brier score of 0.158, demonstrating robust overall performance. The decision tree (DT) model exhibited comparable discriminatory ability, with an AUC of 0.783 (95% CI: 0.661–0.818), and attained the highest recall (0.653) and F1-score (0.603) across all models, reflecting strong classification efficacy.

**Conclusion:**

Among the five evaluated models, CatBoost and DT models showed comparable and favorable overall performance for RA-ILD classification. SHAP analysis based on the CatBoost model identified CA199, CA125, and age as the most important contributors to model prediction. Both models hold promise for RA-ILD risk stratification in clinical practice, although further external validation is warranted.

## Introduction

Rheumatoid arthritis (RA) is a systemic inflammatory disease characterized primarily by symmetric polyarthritis, ultimately resulting in joint destruction and disability. The prevalence of extra-articular symptoms in patients with RA ranges from 17.8 to 40.9% (1), with the common pulmonary manifestation being rheumatoid arthritis-associated interstitial lung disease (RA-ILD). The reported prevalence rates of RA-ILD vary widely, ranging from 4 to 58% in different studies (2, 3). Although most RA-related mortality is associated with cardiovascular disease, pulmonary complications are relatively common and account for 10-20% of the overall mortality (4, 5). Most RA-associated pulmonary involvement is associated with ILD, which is linked to a mortality rate approximately eight times higher than that for RA (6, 7). However, early identification and screening of RA-ILD remain challenging in primary hospitals due to limited diagnostic resources. The early diagnosis and effective management of ILD are, therefore, of crucial importance in improving patient survival rates and quality of life.

High-resolution computed tomography (HRCT) of the chest remains the cornerstone for the diagnosis and evaluation of ILD and pulmonary infections in patients with RA (8). Given that ILD can develop at any stage of RA, serial HRCT scans are often necessary for both the initial diagnosis and subsequent monitoring. This need for repeated imaging, however, raises concerns regarding cumulative exposure to ionizing radiation, and imposes a significant financial burden on patients. Therefore, there is an urgent clinical need for a more convenient, low-risk approach to assist in the identification of ILD in patients with RA.

The development of machine learning (ML) has led to a paradigm shift in medicine, as it provides substantial support for evidence-based clinical decision-making (9, 10). Fundamentally, ML is a computational approach that derives its predictive power from mathematical models trained on data. Its core utility lies in its ability to construct models that learn from data patterns to uncover latent correlations and generate predictive insights (11). In contrast to traditional rule-based algorithms, ML algorithms infer parameters directly from data samples, thus exhibiting superior capability in the identification and analysis of complex patterns. The application of ML in rheumatology enables both pattern recognition and predictive analysis. For instance, ML has been found effective in the identification of immune signatures associated with subtypes of juvenile idiopathic arthritis (12), the prediction of disease trajectories in systemic lupus erythematosus (SLE) (13).

In studies related to rheumatoid arthritis, Qin et al. (14) and Xu et al. (15) developed RA-ILD models using non-routine biomarkers such as KL-6 and IL-6, limiting their application in primary hospitals. Although the logistic regression model proposed by Zhou et al. (16) performed well, it relied on radiographic joint staging. Based on 410 patients with RA, this study constructed a machine learning classification model using only routine clinical and laboratory indicators. This model can be applied in primary hospitals, providing individualized screening evidence for RA patients suspected of ILD, guiding targeted chest HRCT examinations to achieve early and accurate diagnosis, reducing unnecessary CT scans, and lowering the risks of radiation exposure and medical costs.

## Materials and methods

### Data collection

**FIGURE 1:**
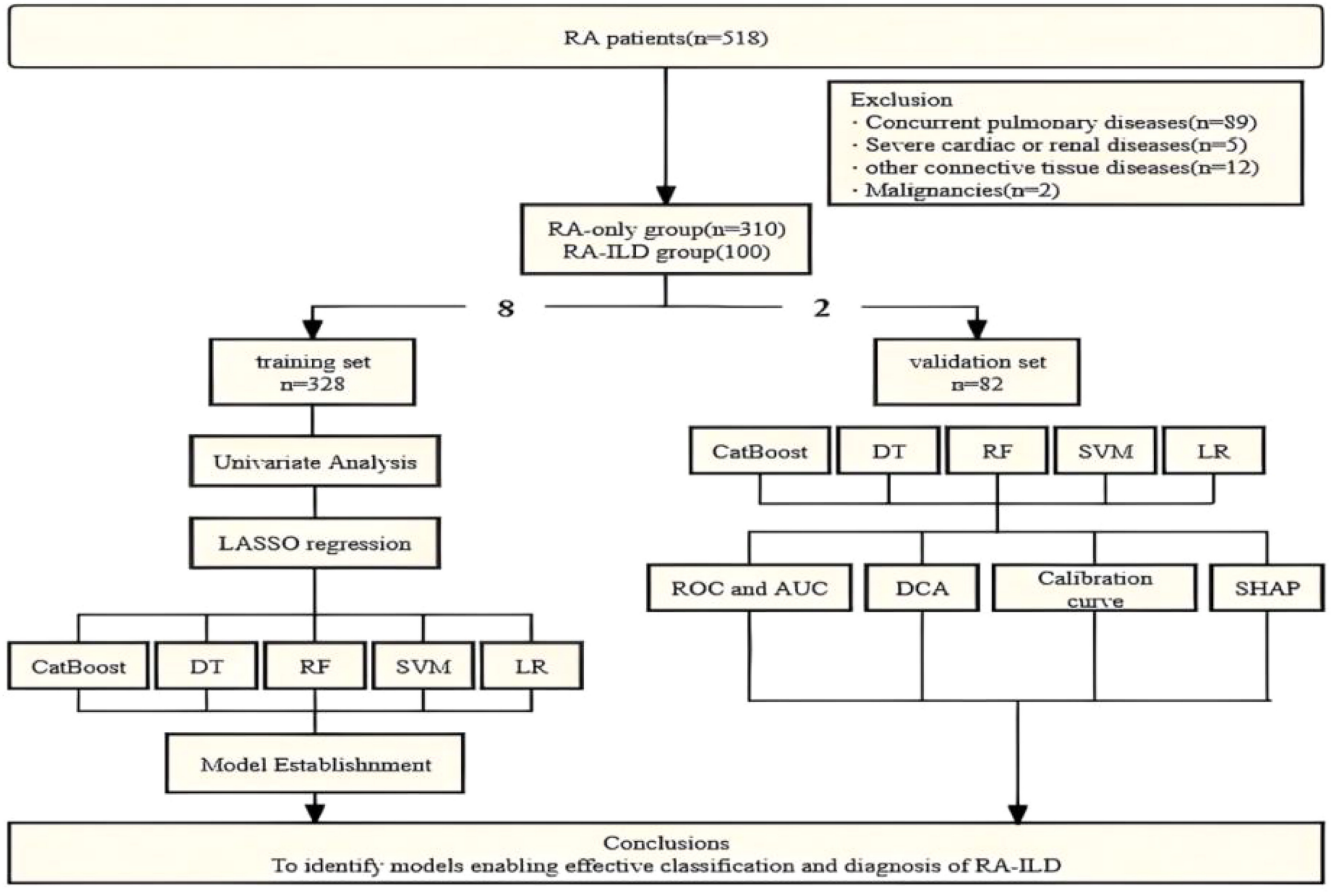
Flowchart of study participant recruitment, feature selection, machine learning model establishment.

Four hundred and ten patients with RA who were diagnosed and treated at the Department of Rheumatology and Immunology, Deyang People’s Hospital, China, between June 2024 and June 2025 were included in the analysis. All patients met the 2010 ACR/EULAR classification criteria for RA. Interstitial lung disease (ILD) diagnosis referred to the diagnostic criteria updated by the American Thoracic Society (ATS)/European Respiratory Society (ERS) in 2013. The following exclusion criteria were applied: (1) Concurrent pulmonary conditions, such as pulmonary infection, bronchial asthma, or chronic obstructive pulmonary disease; (2) Lung diseases caused by physical or chemical factors, including pneumoconiosis; (3) Comorbidities including severe chronic cardiac, hepatic, or renal insufficiency; (4) Coexistence of other connective tissue diseases, hematological disorders, or malignancies; and (5) Pregnancy or lactation as shown in Figure 1. The following data were collected for each patient: (1) Demographic information, including sex, age, disease duration, and smoking history; (2) Clinical data, including number of swollen and tender joints, and visual analog scale (VAS) scores; (3) Serological test results, including the complete blood count, indicators of liver and renal function, C-reactive protein (CRP), erythrocyte sedimentation rate (ESR), immunoglobulins and complement, rheumatoid factor, anti-cyclic citrullinated peptide (CCP) antibodies, carcinoembryonic antigen (CEA), cancer antigens 125 and 199 (CA125, CA199), alpha-fetoprotein (AFP), fibrinogen, (Fg), and D-dime(D-D); (4) Auxiliary examinations, such as chest HRCT.

### Data preprocessing and feature selection

Missing data rates ranged from 0 to 2.4% across all variables. The median imputation method was used to impute missing values. All continuous variables were standardized to a z-score. Binary categorical variables, including sex, smoking history, ANA, anti-CCP antibodies, and Ro52, were converted into numerical format. Given the limited sample size of this study, we prioritized alleviating model overfitting and improving modeling efficiency. First, univariate analysis was performed on 40 candidate variables, and variables with a trend toward significance (*P* < 0.1) were retained for subsequent LASSO regression. The optimal penalty parameter λ in LASSO regression was determined using the 1-standard error (1-SE) criterion, which was applied to reduce feature space redundancy, enhance model performance, and further prevent overfitting. Finally, only features with non-zero coefficients from LASSO regression were retained, yielding a final set of seven key predictors for the development of a machine learning-based classification model for RA-ILD.

### Machine learning models and model validation

The study cohort of 410 participants was randomly divided into a training set (328 cases) and a validation set (82 cases) at a ratio of 8:2. Five machine learning algorithms, CatBoost, LR, SVM, DT, and RF, were used to develop Risk classification modes for RA-ILD. To optimize the performance of the models, hyperparameter tuning was performed with each algorithm through grid searching combined with five-fold cross-validation. Model performance was comprehensively evaluated in terms of accuracy, recall, precision, the F1 score, and AUC values. The ROC curves were plotted with the false positive rate (FPR) on the x-axis and the true positive rate (TPR) on the y-axis. Additionally, calibration curves and the Brier score were utilized to assess predictive performance. Decision curve analysis (DCA) was performed to evaluate the clinical utility of the models.

### Visualization of results and assessment of feature importance

The SHapley Additive exPlanations (SHAP) method was used to assess and visualize the relative contributions of the different features to the output of the representative RA-ILD classification and diagnosis models. This approach quantifies the contribution of each feature to individual classifications and aggregates these values to enable a comprehensive global interpretation of the models. Compared to conventional measures for assessing feature importance, SHAP has the advantage of enabling precise quantification of the influence of each variable as well as the direction of its impact (whether positive or negative). This capability allows for a clinically meaningful interpretation of key features, helping to elucidate their potential roles as either favorable or adverse factors in the context of RA-ILD.

### Statistical analysis

Data were analyzed using the Jupyter environment in Python version 3.12.7. Continuous variables conforming to a normal distribution were presented as mean ± standard deviation (SD) and compared using independent-sample *t*-tests; those with non-normal distribution were presented as median (interquartile range, IQR) and analyzed using the Mann-Whitney U test. Categorical variables were presented as n (%) and analyzed using the chi-square test. Feature selection was performed via univariate analysis followed by LASSO regression. Before model construction, all feature data were standardized using the StandardScaler function from the Scikit-learn library. A *P*-value < 0.05 was considered statistically.

## Results

### Baseline characteristics

A total of 410 patients with RA were included in the study, of whom 106 were male (25.9%) and 304 were female (74.1%). Based on the presence or absence of ILD, the patients were divided into two groups: the RA-only group (*n* = 310) and the RA-ILD group (*n* = 100). The average age of the RA-only group was 59.98 ± 11.36 years, with an average disease duration of 101.60 ± 122.16 months, while the average age of the RA-ILD group was 66.98 ± 8.84 years, with an average disease duration of 110.44 ± 140.80 months. The prevalence of RA-ILD was 24.39% in the overall cohort univariate analysis was performed for 40 variables. The results showed that age, smoking history, WBC, LYMPH, NEUT, EO#, RDW-SD, PCT, ALB, A/G, ALT, AST, LDH, eGFR, IgA, CRP, RF, CCP > 400, ANA, CEA, AFP, CA125, and, CA199 were statistical trend between RA with RA-ILD (*P* < 0.1). As shown in Table 1. These variables with a tendency toward differences were then included in LASSO regression analysis to select the final feature variables for model construction.

**TABLE 1:** Univariate analysis of risk factors associated with RA-ILD.

Feature	RA	RA-ILD	*P*-value
Gender			0.600
Male	78	28	
Female	232	72	
Smoking			0.007
Yes	56	31	
No	254	69	
Ro52			0.61
Positive	39	15	
Negative	271	85	
CCP			0.003
>400	211	83	
≤ 400	99	17	
ANA			0.084
Positive	71	32	
Negative	239	68	
Age (year)	60.00 (53.00, 69.25)	68.00 (61.00, 73.00)	< 0.001
Disease duration (month)	60(12, 120)	42(512, 129)	0.936
DAS28	4.88(4.19, 5.78)	5.00(3.94, 6.08)	0.732
WBC( × 109/L)	6.55(5.36, 8.11)	7.24(5.95, 9.30)	0.006
LYMPH( × 109/L)	1.18(0.90, 1.52)	1.29(0.91, 1.61)	0.082
NEUT( × 109/L)	4.62(3.57, 5.99)	5.12(3.82, 7.07)	0.019
EO#( × 109/L)	0.08(0.04, 0.13)	0.12(0.04, 0.19)	0.005
HGB(g/L)	115.0(104.8, 126.0)	116(105.0, 120.0)	0.491
RDW-SD(fl)	46.60(43.68, 50.63)	48.65(45.15, 51.58)	0.014
RDW-CV(%)	14.10(13.20, 15.20)	14.00(13.23, 15.20)	0.709
PLT( × 109/L)	231.5(180.0, 393.0)	208.5(162.5, 270.8)	0.621
PCT	0.26(0.22, 0.32)	0.24(0.19, 0.29)	0.021
ESR(mm/h)	66.00(40.00, 96.25)	71.50(46.25, 107.0)	0.110
ALB(g/L)	40.55(37.80, 42.70)	38.80(35.13, 42.10)	0.001
GLB(g/L)	28.00(25.10, 31.10)	27.95(25.08, 31.30)	0.910
A/G	1.40(1.30, 1.60)	1.40(1.12, 1.60)	0.065
ALT(U/L)	16.00(12.00, 24.00)	19.00(13.00, 25.75)	0.042
AST(U/L)	21.00(17.00, 27.00)	23.00(18.00, 29.75)	0.058
ALP(U/L)	86.00(72.00, 108.0)	(83.50(67.00, 101.0)	0.217
LDH(U/L)	206.5(177.0, 239.5)	220.0(195.3, 260.8)	0.001
CREA(μmol/L)	60.00(52.58, 70.10)	62.20(52.78, 72.28)	0.336
eGFR(mL/min)	97.75(87.46, 106.1)	94.48(84.70, 100.1)	0.002
IgA(g/L)	2.89(2.10, 3.67)	3.31(2.56, 3.99)	0.005
IgG(g/L)	13.50(11.10, 16.15)	13.70(11.20, 15.95)	0.702
IgM(g/L)	1.30(0.94, 1.80)	1.37(0.90, 1.93)	0.669
C3(g/L)	1.18(1.03, 1.32)	1.18(1, 00, 1.34)	0.540
C4(g/L)	0.27(0.21, 0.33)	0.26(0.21, 0.32)	0.230
CRP(mg/L)	13.99(3.35, 35.68)	19.10(4.34, 68.29)	0.030
RF(IU/mL)	126.0(39.25, 429.5)	219.0(53.35, 740.8)	0.016
Fg(g/L)	4.46(3.61, 5.24)	4.48(3.27, 5.69)	0.801
D-D(mg/L)	1.22(0.70, 3.43)	1.45(0.65, 3.98)	0.840
CEA(ng/mL)	1.04(0.51, 1.70)	1.59(0.88, 2.26)	<0.001
AFP(ng/mL)	2.13(1.36, 3.31)	2.58(1.70, 3.72)	0.013
CA125(U/mL)	13.50(9.98, 17.93)	19.37(10.53, 40.29)	0.001
CA199(U/mL)	12.62(6.28, 23.53)	23.85(12.90, 47.87)	< 0.001

DAS28, disease activity score using 28 joints; WBC, white blood cell count; LYMPH, lymphocyte count; NEUT, neutrophil Count; EO#, eosinophil count; HGB, hemoglobin; RDW-SD, red blood cell distribution width standard deviation; RDW-CV, red blood cell distribution width coefficient of variation; PLT, platelet count; PCT, plateletcrit; ESR, erythrocyte sedimentation rate; ALB, albumin; GLB, globulin; A/G, albumin/globulin ratio; ALT, alanine aminotransferase; AST, aspartate aminotransferase; ALP, alkaline phosphatase; LDH, lactate dehydrogenase; CREA, creatinine; eGFR, estimated glomerular filtration rate; IgA, immunoglobulin A; IgG, immunoglobulin G; IgM, immunoglobulin M; C3, complement 3; C4, complement 4; CRP, C-reactive protein; RF, rheumatoid factor; CCP, cyclic citrullinated peptide antibody; RO52, anti-Ro52; Fg, fibrinogen; D-D, D-dimer; CEA, carcinoembryonic antigen; AFP, alpha-fetoprotein; CA125, cancer antigen 125; CA199, carbohydrate antigen 19-9.

### Feature selection using LASSO regression

**FIGURE 2:**
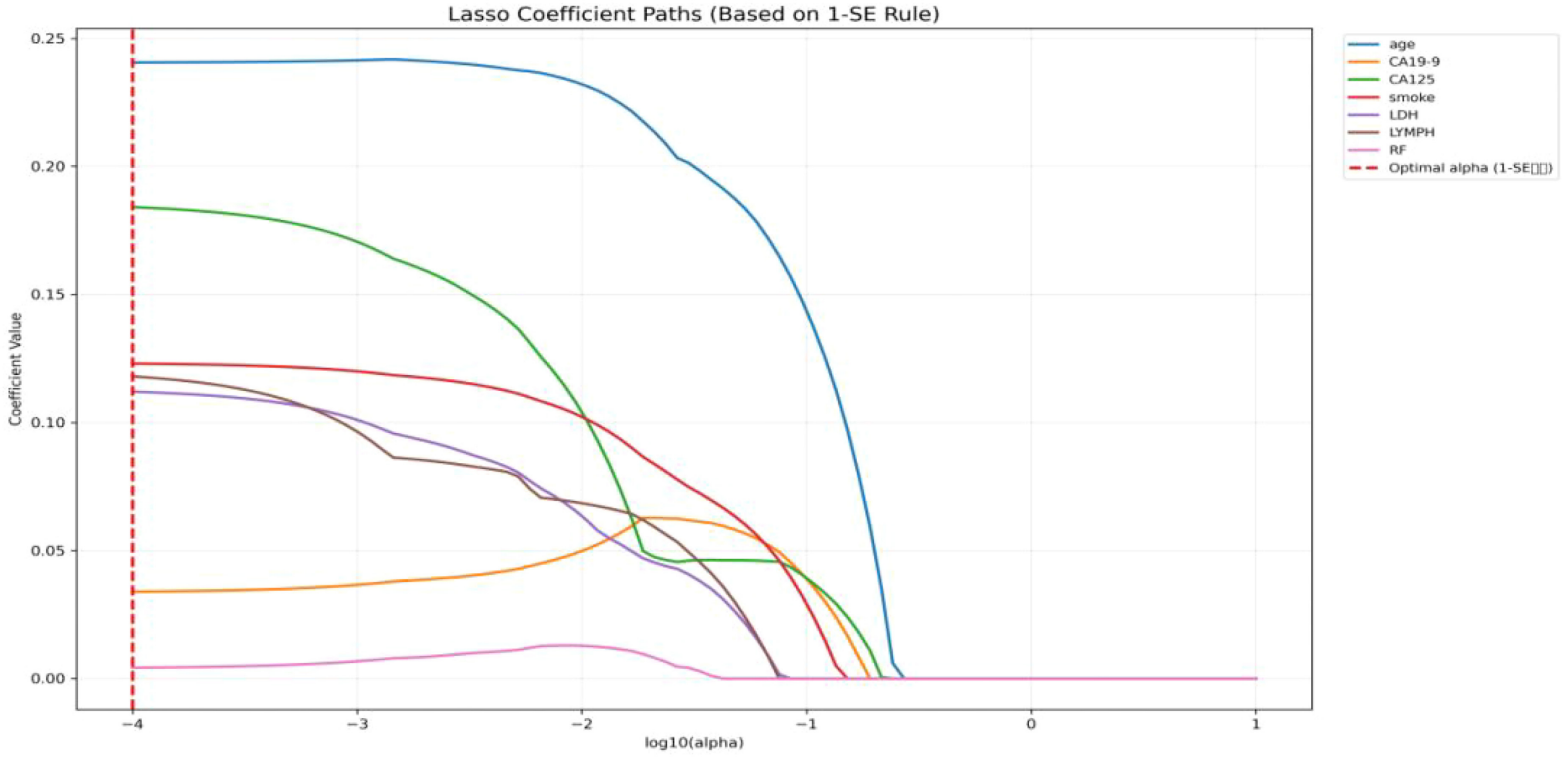
LASSO coefficient paths for the 7 selected features in RA-ILD classification.

Univariate analysis identified 23 variables showing a tendency toward differences between RA and RA-ILD, which were subsequently entered into LASSO regression. This process selected 7 potential feature variables for the classification of RA-ILD: CA199, CA125, age, LDH, LYMPH, smoking history, and RF were positively associated with RA-ILD. These feature variables were subsequently utilized to construct a machine learning-based classification and diagnostic model for RA-ILD. The detailed results are shown in Figure 2.

### Model performance evaluation

**FIGURE 3:**
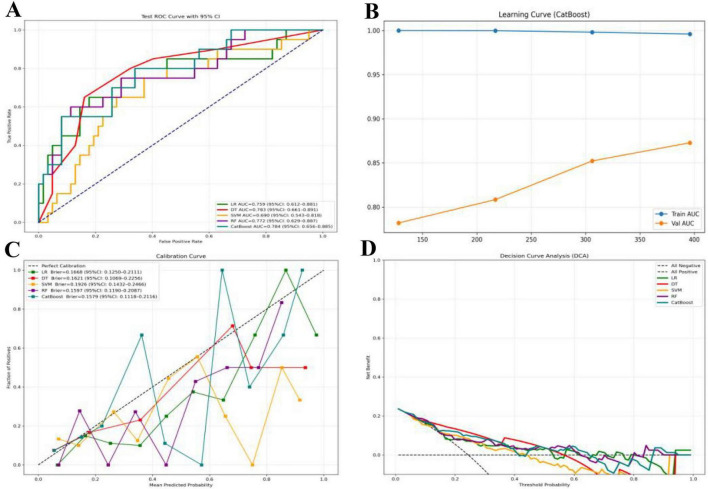
Performance evaluation of the machine learning models for RA-ILD risk prediction. **(A)** Test ROC curves with 95% confidence intervals (CI) for all models. **(B)** Learning curve of the CatBoost model. **(C)** Calibration curves for all models. **(D)** Decision curve analysis (DCA) for all models.

The performance of the five machine learning models (CatBoost, LR, SVM, DT, RF models) was comprehensively evaluated in the validation cohort. After optimization using grid search combined with 5-fold cross-validation, the CatBoost model achieved the highest AUC for RA-ILD classification with optimal hyperparameters (depth = 7, iterations = 150, learning_rate = 0.1). It exhibited a validation set AUC of 0.784 (95% CI: 0.735–0.830) and the lowest Brier score (0.158), with favorable precision, F1-score and recall, indicating superior overall discrimination and calibration. The DT model, with optimized hyperparameters (max_depth = 4, min_samples_split = 30), had a comparable AUC of 0.783 (95% CI: 0.661–0.891) and a Brier score of 0.162, and achieved the highest recall and F1-score across all models, despite its relatively lower stability due to the wider 95% CI. Among the remaining models, random forest (RF) and logistic regression (LR) showed moderate performance, whereas support vector machine (SVM) performed relatively poorly. Detailed performance metrics of all models are summarized in Table 2 and Figure 3A. Given its high AUC and robust generalization capability, the CatBoost model was selected to illustrate model stability and fitting performance. The learning curve of the CatBoost model is presented in Figure 3B. The training AUC remained consistently high (close to 1.0) across all sample sizes, indicating good fitting of the model to the training data. Meanwhile, the validation AUC increased steadily with the expansion of the training sample size, with no signs of overfitting, confirming the model’s strong generalization ability and reliability.

**TABLE 2:** Performance metrics of the risk classification model in the validation set.

Models	AUC	Accuracy	Recall	Precision	F1 score
CatBoost	0.784(0.735–0.830)	0.792(0.707–0.866)	0.554(0.333–0.750)	0.583(0.357–0.792)	0.564(0.353–0.727)
DT	0.783(0.661–0.891)	0.793(0.695–0.878)	0.653(0.435–0.864)	0.571(0.345–0.792)	0.603(0.410–0.759)
RF	0.772(0.723–0.818)	0.793(0.707–0.866)	0.603(0.364–0.813)	0.576(0.350–0.792)	0.583(0.378–0.744)
LR	0.759(0.612–0.881)	0.778(0.683–0.866)	0.600(0.364–0.810)	0.546(0.346–0.760)	0.571(0.372–0.732)
SVM	0.690(0.543–0.818)	0.718(0.622–0.817)	0.404(0.185–0.625)	0.426(0.191–0.654)	0.408(0.194–0.591)

Calibration curves were further constructed to evaluate the consistency between predicted probabilities and actual observed outcomes in Figure 3C. CatBoost and RF exhibited better calibration performance, with Brier scores of 0.1579 (95% CI: 0.1118–0.2116) and 0.1597 (95% CI: 0.1190–0.2087), respectively. Their calibration curves were closely aligned with the perfect calibration line, suggesting reliable and well-calibrated risk predictions. DT showed acceptable calibration, while LR and SVM displayed relatively inferior calibration, with SVM deviating most substantially from the ideal line.

Decision curve analysis (DCA) was performed to assess the clinical utility of the models in Figure 3D. Within a broad range of clinically relevant threshold probabilities, both CatBoost and DT achieved higher net benefits compared with LR, SVM, RF, as well as the “all positive” and “all negative” reference strategies. These findings support the excellent clinical applicability of both models for RA-ILD risk stratification. The models enable accurate identification of high-risk individuals with RA at risk of developing ILD, allowing for targeted chest HRCT evaluation in patients with a high suspicion of RA-ILD to confirm the diagnosis. This strategy effectively reduces the number of unnecessary CT scans by avoiding routine chest HRCT screening for all RA patients, thereby lowering the risk of radiation exposure and healthcare costs.

### Visualization of results: importance ranking and SHAP values

**FIGURE 4:**
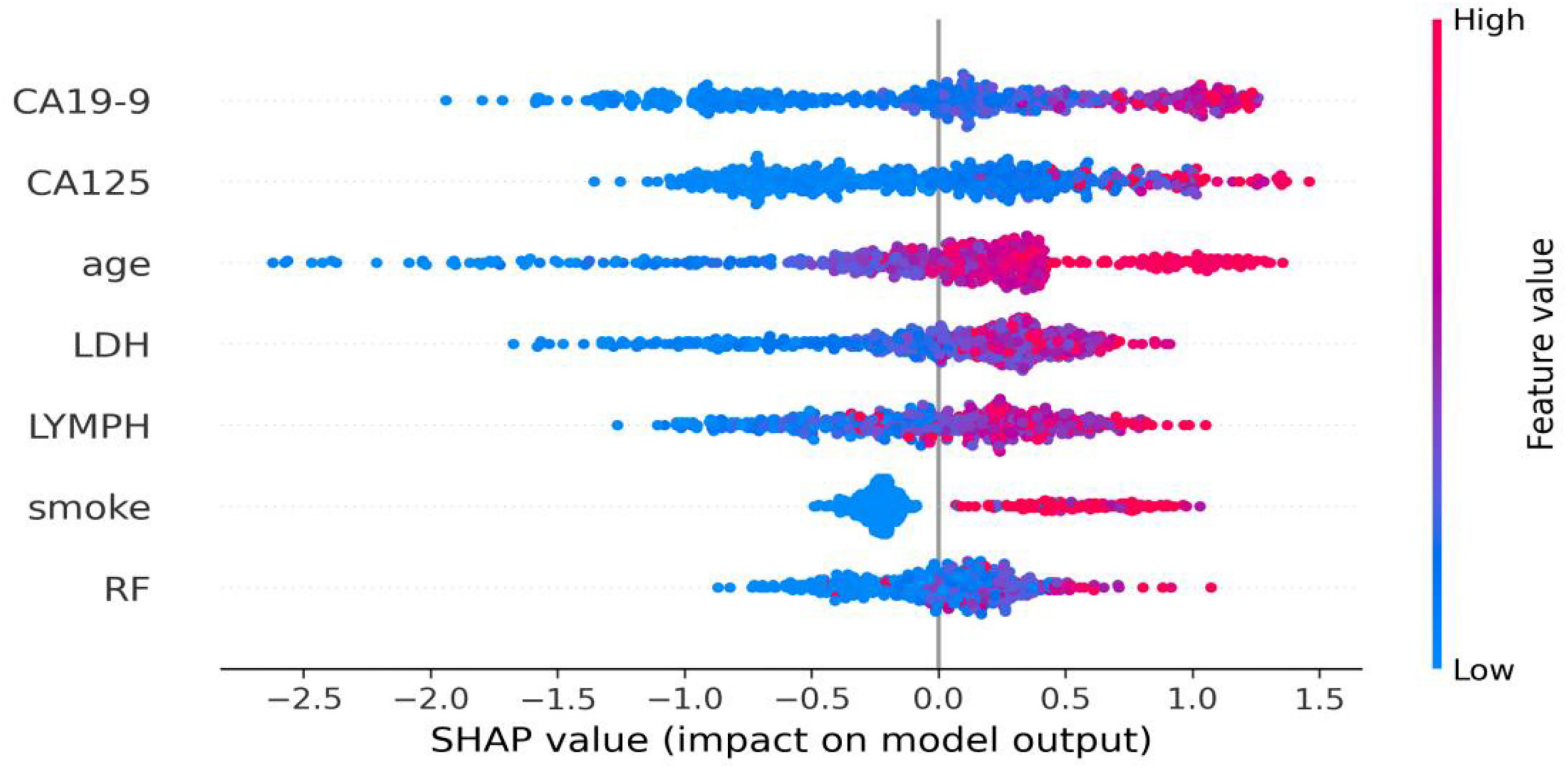
The SHAP summary plot.

The feature importance of the optimal CatBoost model was quantified using SHAP values, visualized in the SHAP summary plot (Figure 4). Each dot represents the SHAP value of an individual sample for a given feature, where a larger absolute SHAP value indicates a greater impact on the model’s prediction. Features are ordered from top to bottom by their mean absolute SHAP value, reflecting their overall contribution to the model. For each feature, a clear trend was observed: red dots (high feature values) clustered on the right (positive SHAP values) and blue dots (low feature values) on the left (negative SHAP values), demonstrating a positive association between the feature and the predicted risk of RA-ILD. The top 5 most impactful features, ranked by their average influence on the model, were CA19-9, CA125, age, LDH, and LYMPH (lymphocyte count). All these key predictors were positively correlated with an increased risk of RA-ILD. Specifically, RA patients with elevated CA19-9 and CA125 levels, older age, higher LDH levels, and higher lymphocyte counts were at significantly greater risk of developing ILD.

## Discussion

ILD is the common extra-articular complication of RA with reported incidence rates ranging from 4 to 58% (2, 3). In this study, the prevalence of RA-ILD was 24.39%. The presence of RA-ILD significantly worsens patient prognosis (17), and the mortality rate among RA patients with ILD is markedly higher than that of those without ILD (2). Therefore, early and accurate assessment of the likelihood of developing ILD in RA patients is crucial for improving outcomes. Currently, clinical diagnosis relies on repeated chest CT scans, which not only pose risks associated with exposure to ionizing radiation but also increase the financial burden on patients. Although several studies have demonstrated the potential diagnostic value of various biomarkers for RA-ILD, these indicators have not been widely adopted in routine clinical practice. To address this issue, this study constructed a classification and diagnostic model for RA-ILD using machine learning based on demographic data and laboratory test results. Relying on routinely accessible indicators, this model is suitable for primary hospitals, which can assist primary care physicians in RA-ILD classification and diagnosis, provide individualized screening evidence for high-risk patients, guide targeted HRCT examinations for early and accurate diagnosis, reduce unnecessary CT scans, lower radiation risks and medical costs, and facilitate the optimization of primary medical resources.

In the present study, univariate analysis identified 23 variables for inclusion in LASSO regression. This procedure selected seven key features for RA-ILD classification: CA19-9, CA125, age, LDH, LYMPH, smoking history, and RF were all positively associated with RA-ILD. These findings are partly consistent with those reported by Zhou et al., who identified older age, smoking, elevated DAS28 scores, higher radiographic joint staging, strong positive CCP status ( > 200 U/mL), and methotrexate therapy as potential predictors for RA-ILD. In contrast, hormone therapy has been observed to act as a protective factor against the development of RA-ILD (16). In contrast, their model required radiographic joint staging in addition to routine clinical and laboratory parameters, which necessitates additional imaging assessment. Qin et al. enrolled 153 RA patients and used three machine learning algorithms to screen diagnostic biomarkers including age, KL-6, D-dimer, and CA19-9 (14). More recently, Xu et al. developed an early prediction model for RA-ILD based on 149 RA patients and four machine learning approaches, with the XGBoost model achieving an AUC of 0.891 by using specific biomarkers such as KL-6, IL-6, and CYFRA21-1 (15). Nevertheless, Qin et al. and Xu et al. relied on non-routine biomarkers and employed relatively small sample sizes, which substantially restricted their universal clinical application. In comparison, the present study enrolled a real-world clinical cohort of 410 RA patients and systematically constructed and compared five mainstream machine learning models. Our model relied entirely on routine clinical and laboratory indicators without extra imaging or specialized biomarker testing, making it more convenient, feasible, and generalizable for widespread application in primary hospitals.

Among the 7 variables included in the classification model constructed in this study, the associations of smoking history, advanced age, and positive RF with RA-ILD were consistent with findings from previous research, further validating the reliability of our model. For instance, a study by Salaffi et al. involving 151 patients identified significant associations between RA-ILD and older age, late-onset RA, smoking, and anti-CCP antibodies (2). Similarly, a study conducted by Gouri Mani Koduri et al. recognized smoking history, older age, RF positivity, and anti-CCP antibodies as key risk factors for the development of RA-ILD (18). Thus, both these studies identify smoking and older age as risk factors for RA-ILD, which is consistent with our results, highlighting the critical roles of smoking and advanced age in the development and progression of RA-ILD. Consequently, it is critically important to screen for ILD in elderly or smoking-positive patients with RA. Patients with RA-ILD often exhibit high titers of RF and anti-CCP antibodies, both of which are higher in the bronchoalveolar lavage fluid than in the serum, suggesting that elevated levels of RF and anti-CCP antibodies may be involved in the process of lung injury in RA-ILD (19). In addition to known risk factors such as older age, smoking history, and RF positivity, this study also showed that CA125, CA199, and LDH are important in the identification of RA-ILD. This finding is consistent with those of Wang T et al., who also observed elevated serum levels of CA125, and CA199 in RA-ILD patients (20, 21). The underlying pathogenesis of ILD is hypothesized to involve sustained injury and repair, apoptosis of epithelial cells, and the formation of fibroblast foci. CA199, CA125 and CEA are documented indicators of epithelial cell proliferation and secretion, which may offer novel insights into the association between tumor markers and ILD (22). Further investigation is warranted to clarify the clinical and biological significance of tumor markers in ILD.

This study has several limitations. First, the retrospective design may have led to both selection and information bias. Second, the data were obtained from a single center, thereby potentially limiting the generalizability of the findings. Meanwhile, the calibration curve of the model showed certain deviations between predicted probabilities and actual observed risks, and decision curve analysis (DCA) indicated that the model provided clinical net benefit only within a relatively narrow threshold range, suggesting suboptimal calibration performance and limited clinical applicability. In addition, the diagnostic model has not been validated in external datasets. Further multicenter and prospective studies are warranted to optimize and validate this predictive model, especially to improve its calibration ability and clinical utility. Although the AUC achieved in the present study was not higher than that in previous reports, our model was specifically developed for RA-ILD screening in primary hospitals. By using only routine clinical and laboratory indicators, the proposed model exhibits greater practicality, accessibility, and generalizability in real-world clinical settings, particularly in grassroots medical institutions.

In summary, this study established a machine learning model for risk classification of RA-ILD using routine clinical data. While the model demonstrated promising performance in internal validation, its clinical utility remains in the preliminary stage. Furthermore, this study identified high-risk populations of RA patients complicated with ILD through common clinical indicators, with subsequent confirmation of diagnosis via chest HRCT examinations. This approach not only enables the early identification of RA patients at high risk of developing ILD but also minimizes unnecessary CT radiation exposure and reduces medical costs, thereby contributing to more efficient and cost-effective clinical management of RA patients. However, the findings of this study still require further external validation in larger, multi-center cohorts. Such validation is crucial to confirm the generalizability of the conclusions and better facilitate the translation of this risk stratification strategy into clinical practice.

## Data Availability

The raw data supporting the conclusions of this article will be made available by the authors, without undue reservation.
